# Supplementary material for: GFAP splice variants fine-tune glioma cell invasion and tumour dynamics by modulating migration persistence
Source: Sci Rep. 2022 Jan 10;12:424. doi: 10.1038/s41598-021-04127-5 (PMC8748899; doi:10.1038/s41598-021-04127-5)
Supplement: Supplementary file 1 — Supplementary Information. [file 41598_2021_4127_MOESM1_ESM.pdf]

Supplementary Figures

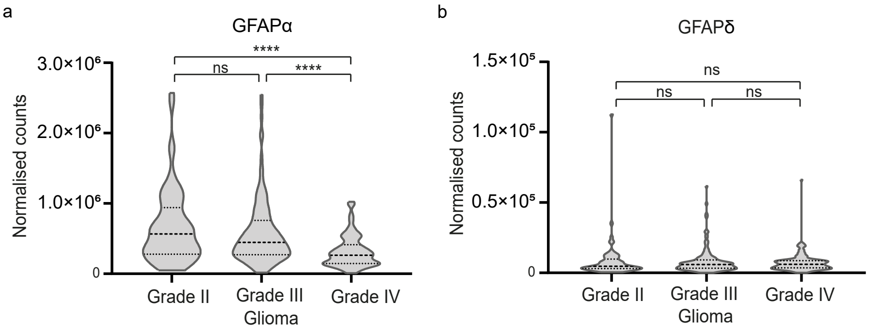

Supp. Fig. 1. GFAP isoform expression in different grades of astrocytoma. Violin plots of GFAPα and GFAPδ levels in tumour samples of grade II (n= 64), grade III (n= 130), and grade IV (n= 153) astrocytoma, obtained from normalised isoform expression data of the TCGA database. GFAPα levels (a) are decreased in grade IV astrocytoma, whereas GFAPδ levels (b) remain constant. Significance was determined using a Kruskal-Wallis test followed by Dunn's multiple comparisons test. The data is shown as mean ± S.E.M, *p < 0.05, **p < 0.01, ***p < 0.001, ****p < 0.0001, ns = not significant.

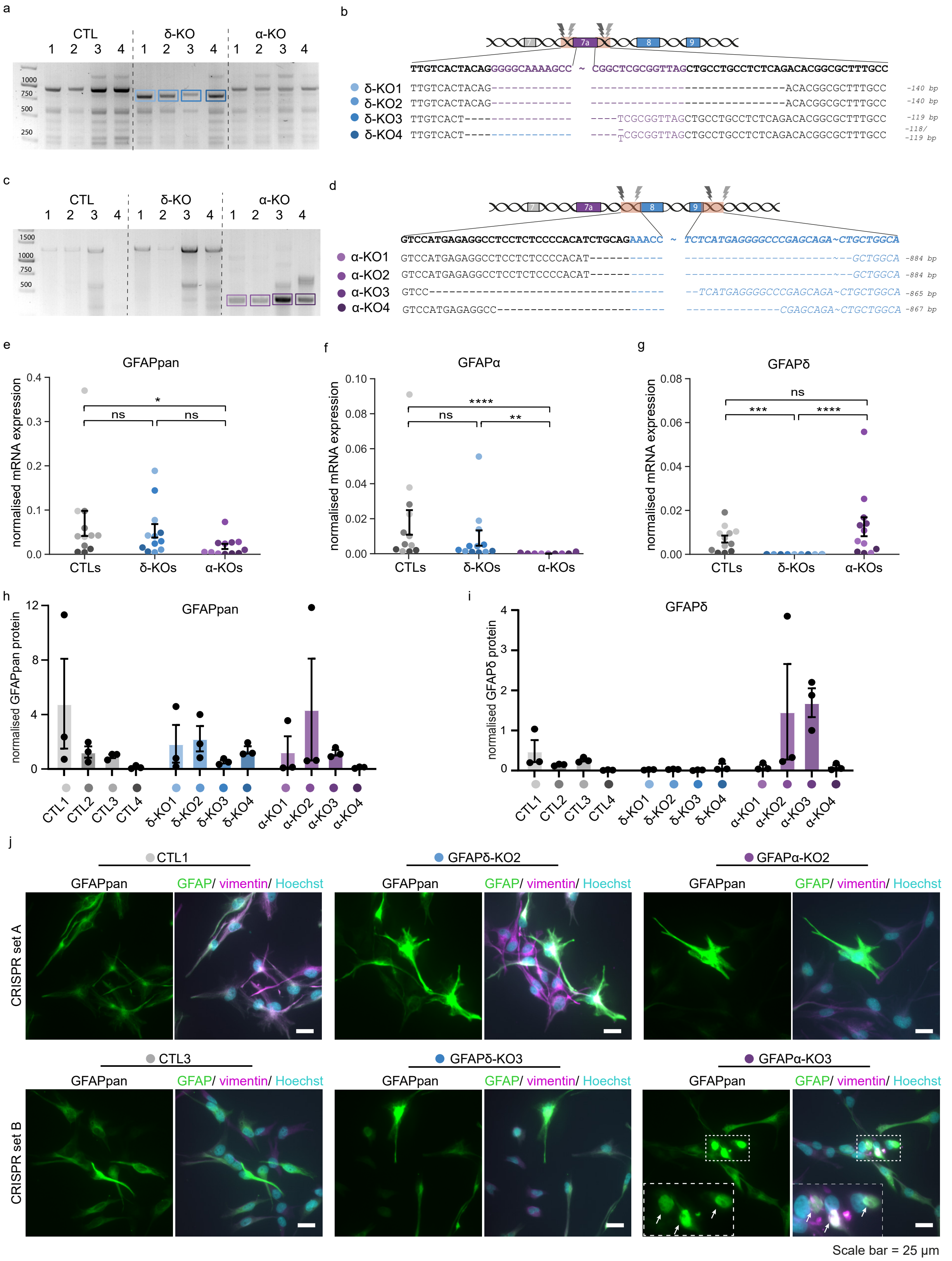

Supp. Fig. 2. Characterisation of the GFAP-modulated cells. (a,b) PCR amplification and sequencing of the GFAP gene around exon 7a. GFAPδ-KO cells have a deletion of 140 bp (GFAPδ-KO 1 and 2, CRISPR δ-set A) or 118/119 bp (GFAPδ-KO 3 and 4, CRISPR δ-set B)

of exon 7a. (c,d) PCR amplification and sequencing of the GFAP gene around exons 8 and 9. GFAPα -KO cells have 884 bp deletion (GFAPα-KO 1 and 2, CRISPR α-set A) or 865/867 bp deletion (GFAPα-KO 3 and 4, CRISPR α-set B) of exons 8 and 9. (e-g) mRNA levels of GFAPpan (e), GFAPα (f), and GFAPδ (h) normalised against GAPDH and AluJ. Deletion of exon 7a (GFAPδ-KO cells) leads to a significant reduction in GFAPδ mRNA levels, but not to a reduction in GFAPα or GFAPpan. Deletion of exons 8 and 9 (GFAPα-KO cells) leads to a significant reduction in both GFAPα and GFAPpan levels, but not in GFAPδ mRNA levels. n= 12 individual experiments per group, derived from 4 clones per condition represented with different colour hues. Significance was determined using a Kruskal-Wallis test followed by Dunn's multiple comparisons test. (h,i) Protein levels of GFAPpan (g) and GFAPδ (h) normalised against GAPDH. (j) The GFAP network in six different cell clones (CRISPR set A: CTL1, GFAPδ-KO3, GFAPα-KO3, CRISPR set B: CTL3, GFAPδ-KO2, GFAPα-KO2) shown with immunofluorescence. GFAP is integrated in the IF network (shown with vimentin) in all cell clones. IF network collapses were occasionally observed in GFAPα-KO3, indicated with white arrows. Scale bar = 25 µm. The data is shown as mean ± S.E.M, *$p < 0.05$, **$p < 0.01$, ***$p < 0.001$, ****$p < 0.0001$, ns = not significant.

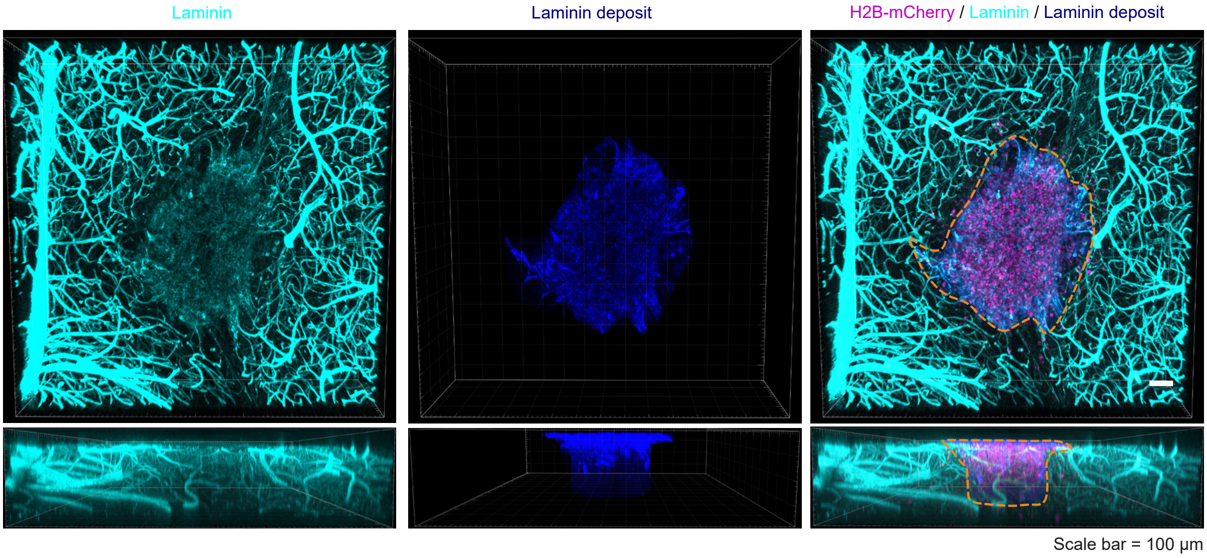

Supp. Fig. 3. Laminin staining can be used to distinguish tumour core from invading cells. Overexposure of laminin reveals background staining that colocalizes with the highest density of H2B-mCherry nuclei at the site of injection. This laminin deposit is used to distinguish cells in the tumour core *versus* cells that invaded the tissue, indicated with the orange dotted line. Scale bar = 100 µm**.**

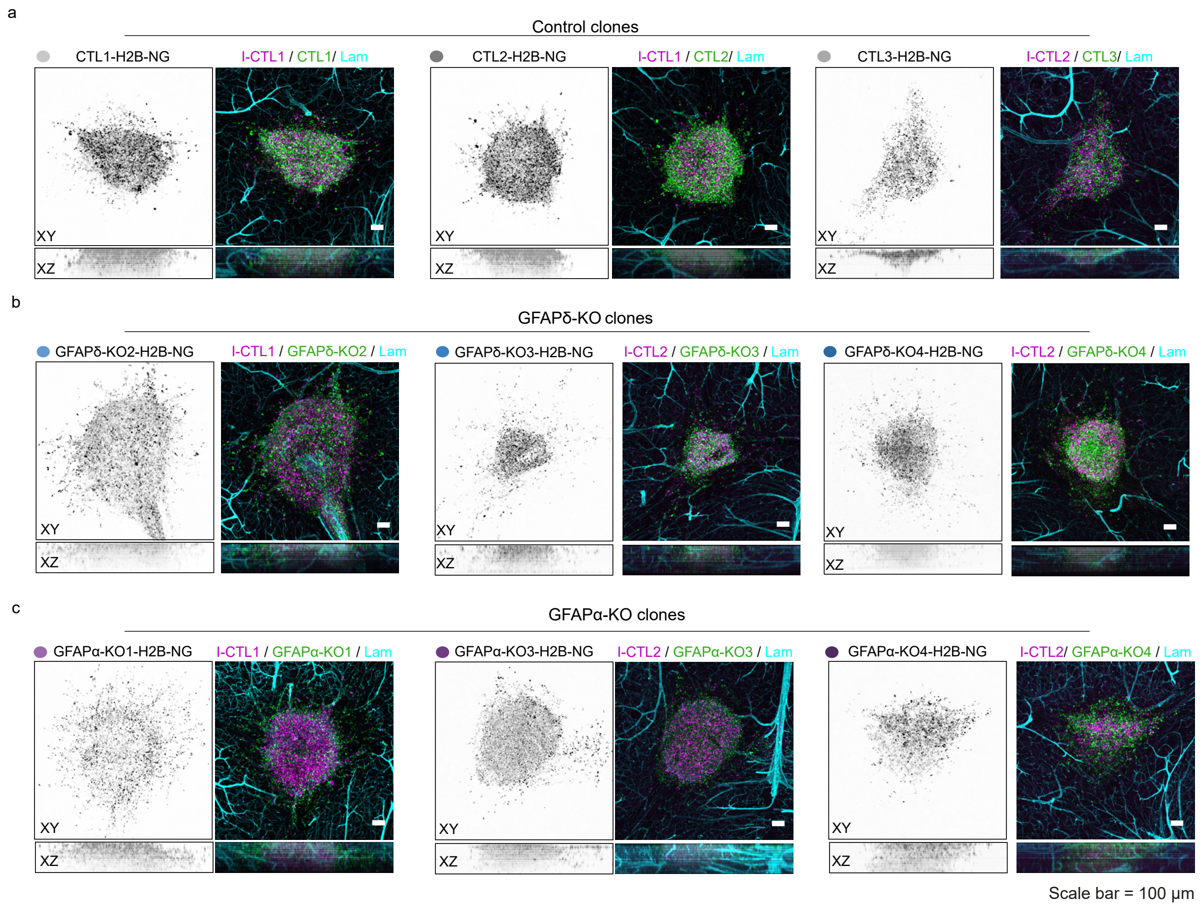

Supp. Fig. 4. Representative images of organotypic slice cultures injected with the different cell clones. Representative images of injected I-CTL-H2B-mCherry cells together with H2B-mNeonGreen expressing CTL clones (a), GFAPδ-KO clones (b) or GFAPα-KO clones (c). Scale bar = 100 µm. NG= mNeonGreen, Lam = laminin.

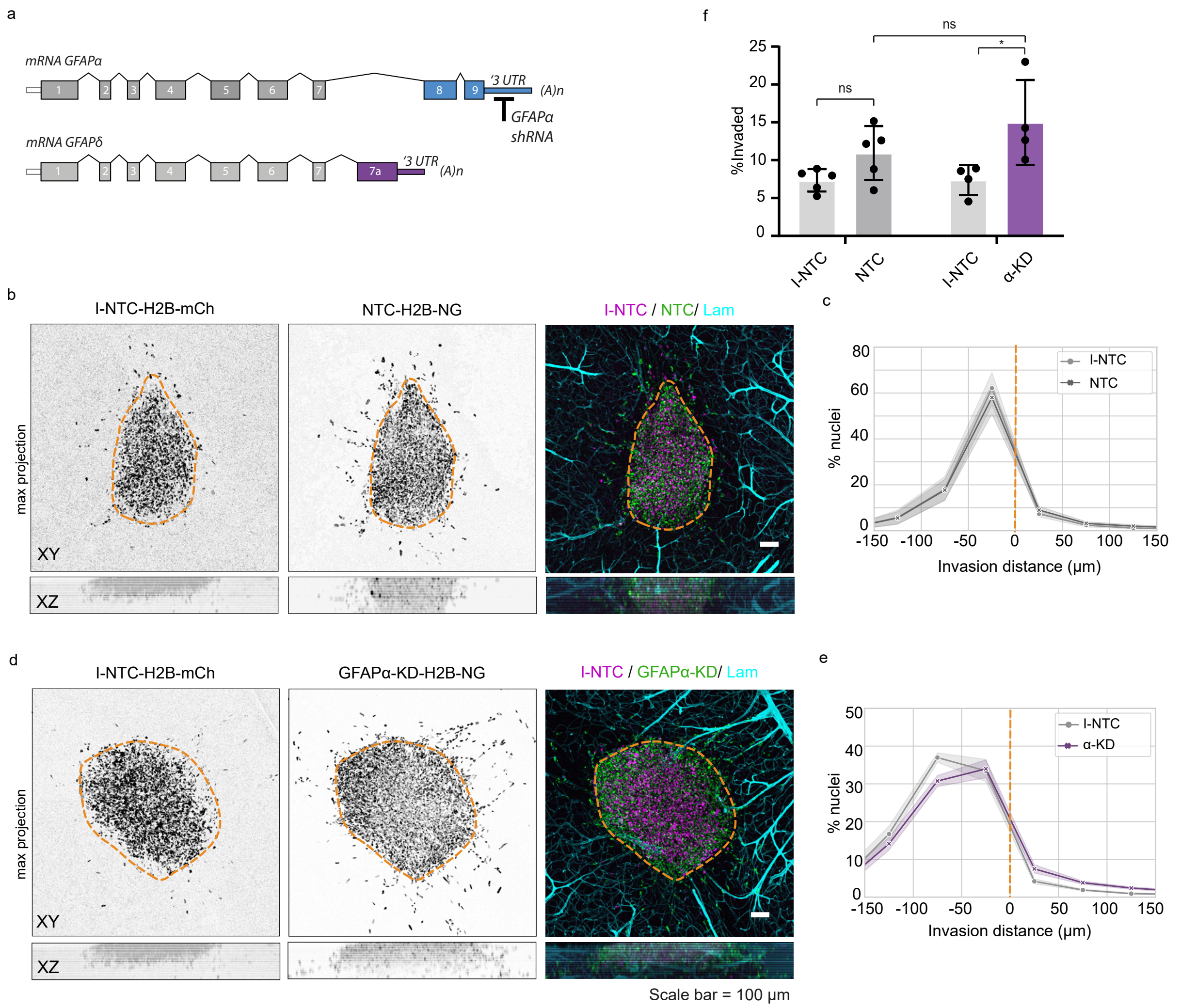

Supp. Fig. 5. Invasion patterns of GFAPα-KD cells. (a) Schematic illustration of GFAPα shRNA target site. (b,c) I-NTC and NTC show similar distribution patterns of nuclei in organotypic brain slices. Histograms show the percentage of nuclei per 50 μm bins, with negative values representing cells within the tumour core and positive values representing cells in the mouse brain tissue (n= 5 independent experiments). (d,e) GFAPα-KD cells show a more diffuse growth pattern in comparison to I-NTC cells and a shift in cell distribution towards the mouse tissue (n=4 independent experiments). (f) Quantification of the percentage of invaded cells. GFAPα-KD cells show higher percentages of cell invasion in comparison to the I-NTCs, but not in comparison to the NTCs. Significance was determined using a two-way ANOVA followed by Tukey's multiple comparisons test. Scale bar = 100 μm. The data is shown as mean ± S.E.M, **p < 0.05, **p < 0.01, ***p < 0.001, ****p < 0.0001, ns = not significant. I-NTC = internal non-targeting control, NTC = non-targeting control, NG = mNeonGreen, mCh = mCherry, Lam = laminin, UTR = untranslated region.

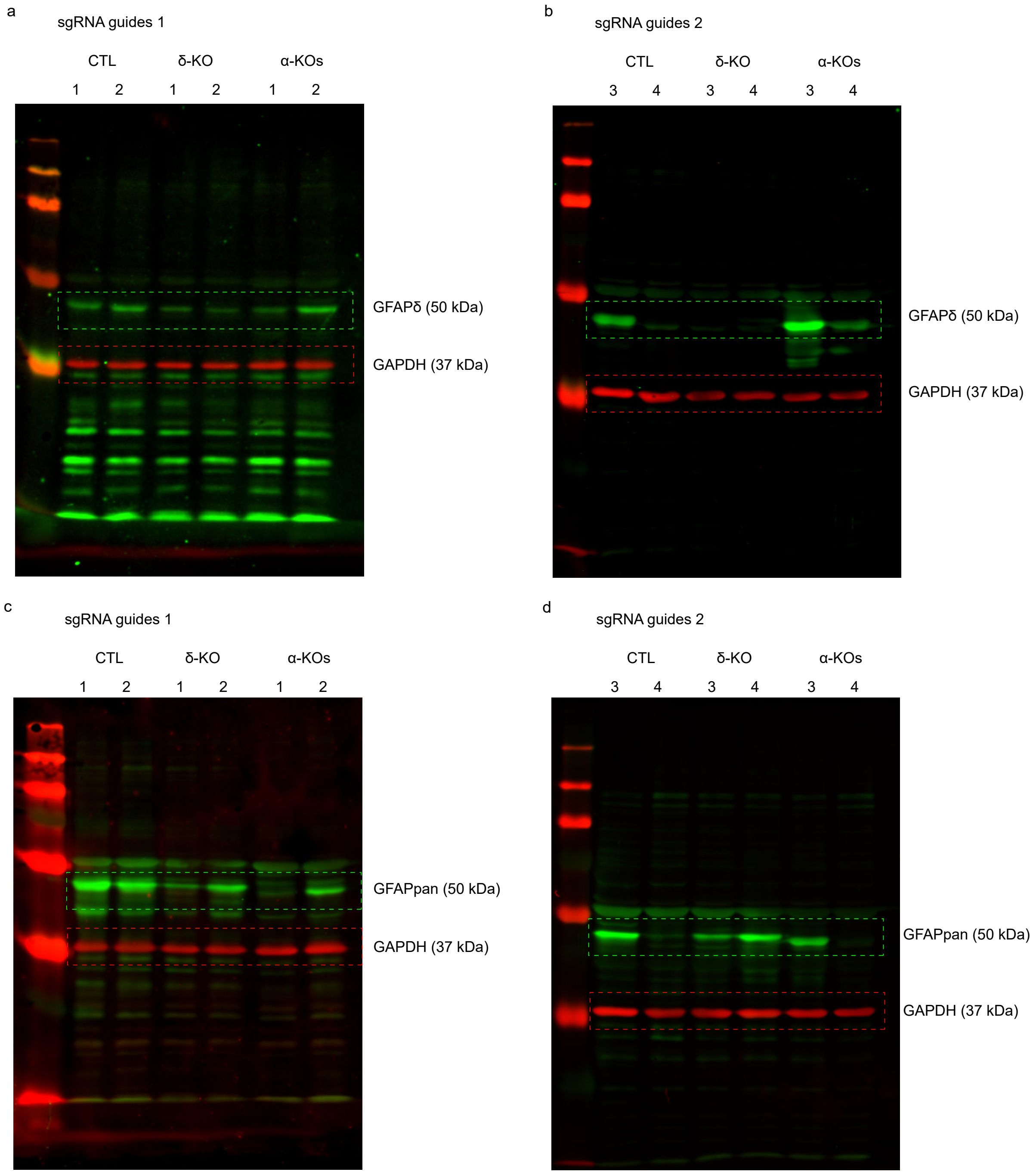

Supp. Fig. 6. Protein levels of GFAPδ and all GFAP isoforms (GFAPpan) in the 12 different cell clones determined with Western blot (a,b) Full-length blots of GFAP and GAPDH. Single blots were stained with primary antibodies rabbit anti-GFAPδ and mouse anti-GAPDH and with secondary antibodies donkey anti-rabbit IRDye800 and donkey anti-mouse AF647 (Table S2). (c,d) Full-length blots of GFAP and GAPDH. Single blots were stained with primary antibodies rabbit anti-GFAPpan and mouse anti-GAPDH and with secondary antibodies donkey anti-rabbit IRDye800 and donkey anti-mouse AF647 (Supp. Table 2). All antibodies were characterised in Moeton et al., 2016.

<u>Supplementary Tables</u>

Supp. Table 1. Overview of oligonucleotides

Name	Target	Oligonucleotide 1 (5' > 3')	Oligonucleotide 2 (5' > 3')
CRISPR-guides			
GFAPα-KO sgRNAs 1	*GFAP* gene intron 7	CACCGGGCTGGTTT CTGCAGATGTG	AAACCACATCTGCA GAAACCAGCCC
	GFAP gene '3 UTR exon 9	CACCGGATAGTTGC TCCGCCTCTGC	AAACGCAGAGGCG GAGCAACTATCC
GFAPα-KO sgRNAs 2	*GFAP* gene intron 7	CACCGCAGGCTGGT TTCTGCAGATG	AAACCATCTGCAGA AACCAGCCTGC
	GFAP gene '3 UTR exon 9	CACCGCTCGGGCC CCTCATGAGACG	AAACCGTCTCATGAG GGGCCCGAGC
GFAPδ-KO sgRNAs 1	*GFAP* gene intron 6	CACCGTAACTGCTTG TCACTACAGG	AAACCCTGTAGTGA CAAGCAGTTAC
	GFAP gene intron 7	CACCGGGCAAAGCG CCGTGTCTGAG	AAACCTCAGACACG GCGCTTTGCCC
GFAPδ-KO sgRNAs 2	*GFAP* gene intron 6	CACCGTTTAACTGCT TGTCACTACA	AAACTGTAGTGACAA GCAGTTAAAC
	GFAP gene intron 7	CACCGAATGGAACG CCGCCGGCTCG	AAACCGAGCCGGCG GCGTTCCATTC
shRNA-guides			
shRNA GFAPα	GFAPα transcript nt 2674–2694	CCCTTCTTACTCACA CACAAA	na
NTC	na	CAACAAGATGAAGAGC ACCAA	na
PCR primers			
GFAPα	*GFAP* gene 44,907,012-44,908,284	TAGGCTCTCTCTGCTC GGTT	GAGGGCGATGTAGTAGGT GC
GFAPδ	*GFAP* gene 44,910,531-44,909,596	GTTGCTCCAGACTGGG ACTG	CATTTCAGGGCCAATGCA AG
qPCR primers			
AluJ	Repeat sequence AluJ	CAACATAGTGAAACCC CGTCTCT	GCCTCAGCCTCCCGAGTA G
GAPDH	GAPDH transcript	TGCACCACCAACTGCT TAGC	GGCATGGACTGTGGTCAT GA
GFAPα	GFAP transcript 1 (α) 2776-2890	CCCACTCTGCTTTGACT GAGC	CCTTCTTCGGCCTTAGAG GG
GFAPδ	GFAP transcript 2 (δ) 1167 - 1263	TCCAACCTGCAGATTC GAGG	TTGGTATAACTCGTATTGT GAGGCTT
GFAPpan	GFAP transcript 1,2 453 - 551	GACCTGGCCACTGTGA GG	GGCTTCATCTGCTTCCTGT C

Abbreviations: AluJ = Alu element Jurka, GAPDH = Glyceraldehyde 3-phosphate dehydrogenase, GFAP = glial fibrillary acidic protein, qPCR = quantitative polymerase chain reaction, sgRNA= single guide RNA, shRNA= short hairpin RNA, PCR = polymerase chain reaction, UTR= untranslated region

Supp. Table 2. List of antibodies

Antibody	Product number, Company	(WM)-IF	WB
Chicken anti-vimentin	AB5733, Chemicon, Temecula, CA, USA	1:1500	-
Mouse anti-GAPDH	MAB374, Chemicon, Temecula, CA, USA	-	1:2000
Rabbit anti-GFAP	#Z0334, Dako (Agilent), Santa Clara, CA, USA	1:1000	1:50000
Rabbit anti-GFAPδ	Manufactured in house. Bleeding date: 27-11-2003 (purified in 2004)	-	1:2000
Rabbit anti-laminin	L9393, Sigma Aldrich, St Louis, MO, USA	1:1000	-
Donkey anti-chicken Cy3	703-175-155, Jackson Immuno Research, West Grove, PA, USA	1:1000	-
Donkey anti-mouse AF647	703-606-150, Jackson Immuno Research, West Grove, PA, USA	-	1:2000
Donkey anti-rabbit Cy3	711-166-152, Jackson Immuno Research, West Grove, PA, USA	1:1000	-
Donkey anti-rabbit AF647	711-606-152, Jackson Immuno Research, West Grove, PA, USA	1:1000	-
Goat anti-rabbit IRDye800	926-32211, LI-COR, 4647 Superior Street Lincoln, NE, USA	-	1:5000

Abbreviations: AF= Alexa Fluor, GAPDH= Glyceraldehyde 3-phosphate dehydrogenase, GFAP = glial fibrillary acidic protein
